# DDX3X promotes the biogenesis of a subset of miRNAs and the potential roles they played in cancer development

**DOI:** 10.1038/srep32739

**Published:** 2016-09-02

**Authors:** Luqing Zhao, Yitao Mao, Yuelong Zhao, Yanong He

**Affiliations:** 1 Department of Pathology, Xiangya Hospital, Central South University, Changsha, Hunan 410008, China; 2 Department of Pathology, School of Basic Medical Science, Xiangya School of Medicine, Central South University, Changsha, Hunan 410013, China; 3 Department of Dermatology, Xiangya Hospital, Central South University, Changsha, Hunan 410008, China; 4 Hunan Key Laboratory of Skin Cancer and Psoriasis, Xiangya Hospital, Central South University, Changsha, Hunan 410008, China; 5 Department of Radiology, Xiangya Hospital, Central South University, Changsha, Hunan 410008, China; 6 School of Computer Science and Engineering, South China University of Technology, Guangzhou, Guangdong 510640, China

## Abstract

DDX3X, located on the X-chromosome, belongs to the DEAD-box RNA helicase family and acts as a key RNA-binding protein to exert its regulatory functions in various biological processes. In this paper, knock-down the expression of DDX3X can affect a subset of miRNA expression levels, especially for miR-1, miR-141, miR-145, miR-19b, miR-20a and miR-34a. Through adopting the immunoprecipitation (IP), RNA immunoprecipitation (RIP), dual luciferase reporter assays, we illustrate that DDX3X could interact with Drosha/DGCR8 complex, elevate the processing activity of Drosha/DGCR8 complex on pri-miRNAs, and increase mature miRNA expression levels. For the studies of potential roles and biological functions of DDX3X-dependent miRNAs and their downstream target genes in multiple cancers, we use the primary data from The Cancer Genome Atlas (TCGA), Ingenuity Pathway Analysis (IPA) and several miRNA target prediction databases, to systematically analyze the expression levels of DDX3X-dependent miRNAs in almost 14 kinds of cancers versus normal tissues, and the essential biological functions for their putative downstream target genes. All these findings will provide us novel insights and directions for thoroughly exploring the regulatory mechanisms of miRNA biogenesis, and shed light on effectively searching the clinical significances and biological roles of DDX3X-dependent miRNAs and their target genes in cancer development.

DDX3, a key family member of DEAD-box RNA helicase, exerts essential roles in multiple biological processes and the development of various cancers^1^,^2^. Based on the differences of gene location, DDX3X is located on the X-chromosome bands p11.3–11.23 region, while DDX3Y is located in the azoospermia factor a (AZFa) region of the Y-chromosome^3^. These two DDX3 homologs share 92% similarity in protein sequence identity, and encodes for a 662- or 661-amino acid polypeptide depending on mRNA alternative splicing^4^. Due to the specialized role of DDX3Y in male fertility, people usually focus their studies on DDX3X and refer DDX3 to DDX3X.

Multiple papers published in recent years have demonstrated the key roles of DDX3 in cancer development. For example, in colorectal cancer, DDX3 could enhance oncogenic KRAS-induced tumor invasion via the β-catenin/ZEB1 axis^5^, or promote tumor invasion through the CK1ε/Dvl2 axis^6^. Meanwhile, inhibition of DDX3 expression by a small molecule inhibitor, such as RK-33, could effectively suppress the activity of Wnt/β-catenin signaling and therefore enable DDX3 as a promising therapeutic target for a subset of colorectal cancers^7^. In breast cancer, using a ring-expanded nucleoside analogue (REN)—NZ51, to inhibit the helicase activity of DDX3, it could significantly suppress the viability and motility of breast cancer cells^8^. Moreover, in breast epithelial cells, the expression of DDX3 is directly modulated by HIF-1α in its promoter region^9^. And in invasive breast cancer, the expression of DDX3 is correlated with over-expression of HIF-1α and its downstream genes CAIX, GLUT1 and several hypoxia related genes, suggesting a distinct function of DDX3 under hypoxic conditions and supporting the oncogenic role for DDX3 in breast carcinogenesis^10^. Besides these two types of cancer, DDX3 also exerts its significantly key roles in various cancers, such as lung cancer^11^, hepatocellular carcinoma^12^, oral squamous cell carcinoma^13^, Ewing sarcoma^14^, glioblastoma multiforme^15^, gallbladder carcinoma^16^ and so on.

miRNA is a cluster of small non-coding RNA molecule, usually 22 nucleotides in length, and have been comprehensively studied in recent years^17^,^18^. Undoubtedly, the regulatory networks and biological effects of miRNA downstream targets are worthwhile doing lots of research works, and it can help us to explore new functions and signaling pathways for miRNA. However, investigating miRNA upstream biogenesis mechanisms seem more attractive and essential. As the regulators of miRNA biogenesis process could efficiently control a subset of miRNAs expression and affect much bigger downstream targets networks to exert their functions. In the modulation process of miRNA biogenesis, DEAD-box RNA helicase family members, especially the DDX5 (p68) and DDX17 (p72) have played an important role^19^. Specifically, p68 and p72 can act as components and cofactors in Drosha/DGCR8 complex (microprocessor complex), so as to promote the cleavage activity of Drosha on a subset of pri-miRNAs to pre-miRNAs^20^. Meanwhile, p68 and p72 can also interact with other accessory factors, such as p53, SMAD, ERα, and mediate their functions in regulating Drosha/DGCR8 microprocessor activity and specificity, positively or negatively modulating miRNA biogenesis process^21^,^22^.

As DDX3X and DDX5, DDX17 belong to the same family and share the most part of similarity in their motif structures and sequences, so the regulatory roles of DDX5 and DDX17 in miRNA biogenesis have enlightened us to explore the role of DDX3X in miRNA biogenesis and its downstream biological functions in cancer development. Right now, there is rare study made on DDX3X and miRNA biogenesis, so in this article we will deeply discuss this novel mechanism and analyze its potential functions in cancer.

## Results

### DDX3X affects the change of miRNA expression profile

**Figure 1:**
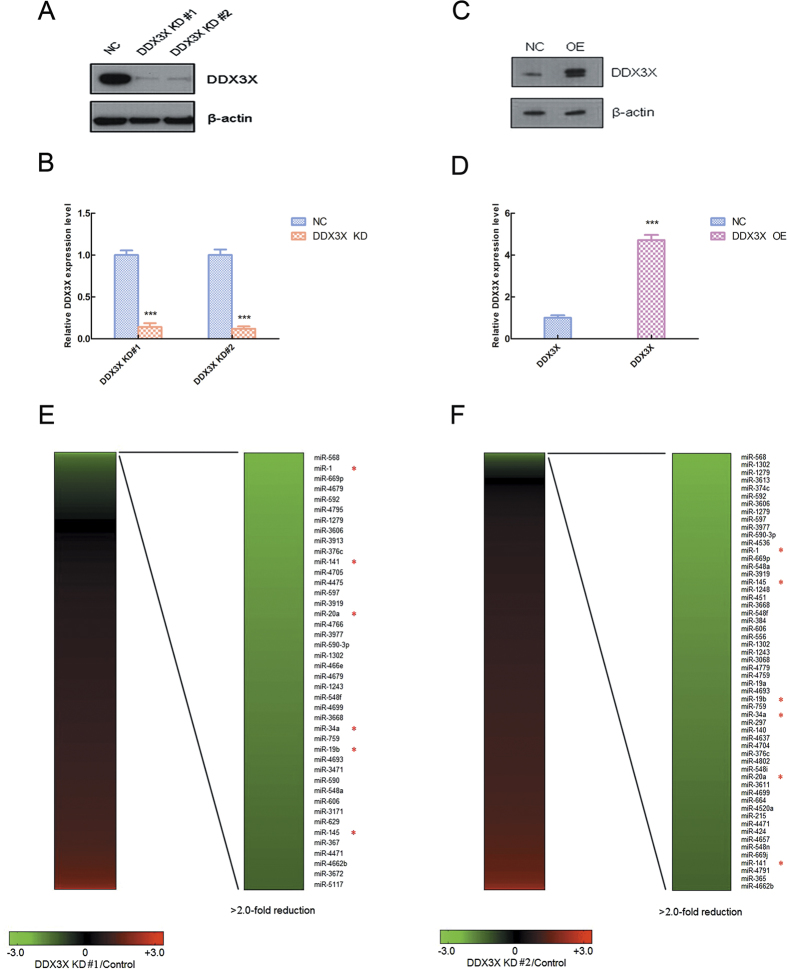
DDX3X affects the change of miRNA expression profile. (**A**) The DDX3X knock-down efficiency of DDX3X KD#1 and KD#2 cell lines tested by western blot. The uncropped full-length blots were presented in Figure S3. (**B**) The relative DDX3X mRNA expression level in DDX3X KD#1 and KD#2 cell lines tested by RT-PCR. The asterisks (***) indicate a significant difference (*p* < 0.001). (**C**) The DDX3X over-expression efficiency of DDX3X OE cell line tested by western blot. The uncropped full-length blots were presented in Figure S3. (**D**) The relative DDX3X mRNA expression level in DDX3X OE cell line tested by RT-PCR. The asterisks (***) indicate a significant difference (*p* < 0.001). (**E**,**F**) The heatmaps for DDX3X KD#1 and KD#2 cell lines compared with negative control cell lines. Red indicated up-regulated miRNAs and green indicated down-regulated miRNAs. The more than 2-fold reduction down-regulated miRNAs overlapped in two heatmaps were marked with a red asterisk (*).

Firstly, we used two kinds of pGIPZ shRNA lentiviral vectors (Thermo Scientific) and their negative control (NC) vectors to infect the U2OS cell lines, so as to knock down the expression of DDX3X and establish the DDX3X stably knock-down (KD) cell lines. Meanwhile, we also used the pCDH cDNA cloning and lentiviral expression vector (System Biosciences) and its negative control (NC) vector to infect the U2OS cell lines, so as to over-express the DDX3X expression and establish the DDX3X stably over-expression (OE) cell lines. Then, we used the western blot and RT-PCR to test the KD and OE efficiency in U2OS cell lines. The data shown that the DDX3X expression levels (protein levels and mRNA levels) were significantly down-regulated in DDX3X KD#1 and KD#2 cell lines (***p < 0.001) (Fig. 1A,B) and up-regulated in DDX3X OE cell lines compared with NC groups (***p < 0.001) (Fig. 1C,D).

Next, we used two DDX3X stably KD cell lines DDX3X KD#1 and KD#2 to do the miRCURY LNA^™^ miRNA array (Exiqon) and screened the profiles of miRNA expression level change when DDX3X expression level is down-regulated. As we wanted to explore the role of DDX3X in miRNA biogenesis, so in this paper we only focused on the down-regulated miRNA profiles and drawn heatmaps for more than 2-fold reduction miRNAs. Compared with two DDX3X KD heatmaps, we can find that there were six miRNAs overlapped in two heatmaps and marked with red asterisk, such as miR-1, miR-141, miR-145, miR-19b, miR-20a and miR-34a (Fig. 1E,F). So in the next part, we will focus on these six miRNAs and do more further researches.

### DDX3X interacts with Drosha/DGCR8 complex and affects the pri-miRNA and mature miRNA expression levels

**Figure 2:**
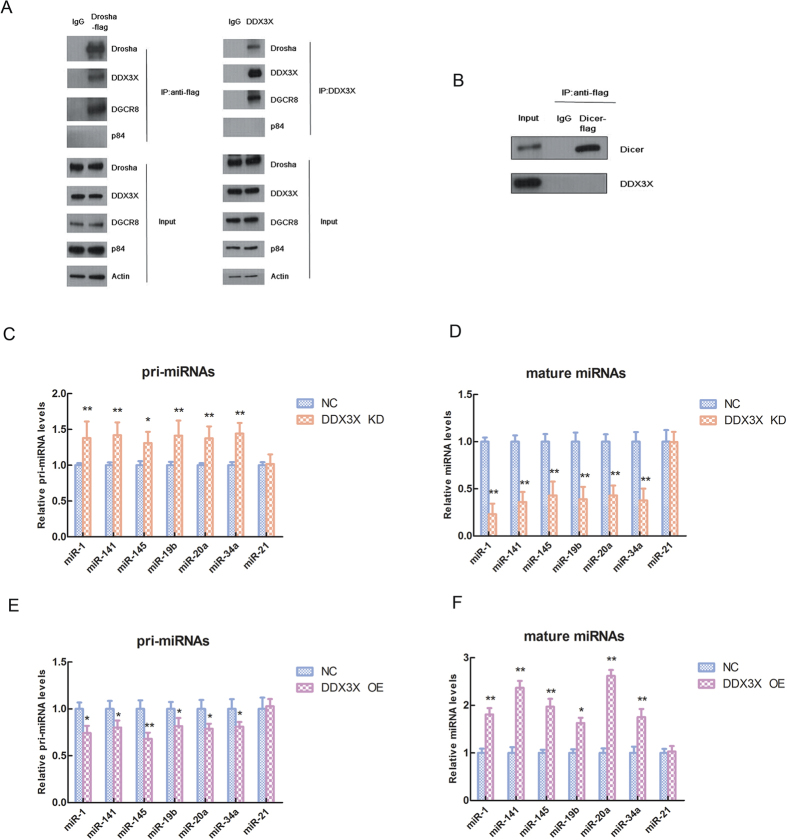
DDX3X interacts with Drosha/DGCR8 complex and affects the pri-miRNA and mature miRNA expression levels. (**A**) The interactions between DDX3X and Drosha complex tested by immunoprecipitation (IP) experiment. The uncropped full-length blots were presented in Figure S3. (**B**) The interactions between DDX3X and Dicer tested by immunoprecipitation (IP) experiment. The uncropped full-length blots were presented in Figure S3. (**C**) Relative pri-miRNA expression levels of DDX3X-dependent miRNAs (miR-1, miR-141, miR-145, miR-19b, miR-20a and miR-34a) in DDX3X KD cell lines compared with negative control (NC) cell lines. The DDX3X-independent miRNA (miR-21) was used as an internal control. The asterisk(s) (*^,^**) indicate a significant difference (*p* < 0.05, *p* < 0.01) respectively. (**D**) Relative mature miRNA expression levels of DDX3X-dependent miRNAs in DDX3X KD cell lines compared with NC cell lines. The DDX3X-independent miRNA (miR-21) was used as an internal control. The asterisks (**) indicate a significant difference (*p* < 0.01). (**E**) Relative pri-miRNA expression levels of DDX3X-dependent miRNAs in DDX3X OE cell lines compared with NC cell lines. The DDX3X-independent miRNA (miR-21) was used as an internal control. The asterisk(s) (*^,^**) indicate a significant difference (*p* < 0.05, *p* < 0.01) respectively. (**F**) Relative mature miRNA expression levels of DDX3X-dependent miRNAs in DDX3X OE cell lines compared with NC cell lines. The DDX3X-independent miRNA (miR-21) was used as an internal control. The asterisk(s) (*^,^**) indicate a significant difference (*p* < 0.05, *p* < 0.01) respectively.

We used HEK293 cell line as a model and transfected this cell with Drosha-flag vector. Then we adopted the flag antibody and carried out the immunoprecipitation (IP) experiment to pull-down the protein complex which is combined with Drosha. The data shown that the DDX3X expression could be tested in the system, and it indicated that DDX3X has an interaction with Drosha complex. Whereas, we used the DDX3X antibody to do the IP experiment reversely and pull-down the protein binding with DDX3X. We have clearly tested the Drosha expression in the DDX3X protein complex (Fig. 2A). All these data demonstrated that DDX3X and Drosha could have an interaction with each other. In this experiment, we used the DGCR8 and p84 as positive control and negative control respectively. Meanwhile, we checked the Dicer-IP to test the interaction between Dicer and DDX3X. However, we couldn’t test the DDX3X expression in the Dicer protein complex (Fig. 2B) and it hinted that DDX3X and Dicer didn’t have an interaction in this system.

In the next part, we used the RT-PCR to double check the miRNA array data and test the pri-miRNA and mature miRNA expression levels of six significantly altered miRNAs (miR-1, miR-141, miR-145, miR-19b, miR-20a and miR-34a) in DDX3X KD and OE cell lines respectively. The data indicated that the pri-miRNAs expression levels were significantly up-regulated and the mature miRNAs expression levels were significantly down-regulated in DDX3X KD cell lines compared with negative control (NC) group (*p < 0.05, **p < 0.01) (Fig. 2C,D) and it demonstrated that knock-down of DDX3X expression could inhibit miRNA biogenesis process and down-regulate mature miRNA expression levels. Conversely, in DDX3X OE cell lines, the data showed that the pri-miRNAs expression levels were significantly down-regulated and the mature miRNAs expression levels were significantly up-regulated compared with NC group (*p < 0.05, **p < 0.01) (Fig. 2E,F) and it indicated that over-expression of DDX3X could promote miRNA biogenesis process and up-regulate mature miRNA expression levels. In these experiments, we used the DDX3X independent miRNA (miR-21) as an internal control to make sure the quality of the system.

### DDX3X facilitates pri-miRNAs binding to Drosha/DGCR8 complex and elevates the processing activity of Drosha/DGCR8 complex on pri-miRNAs

**Figure 3:**
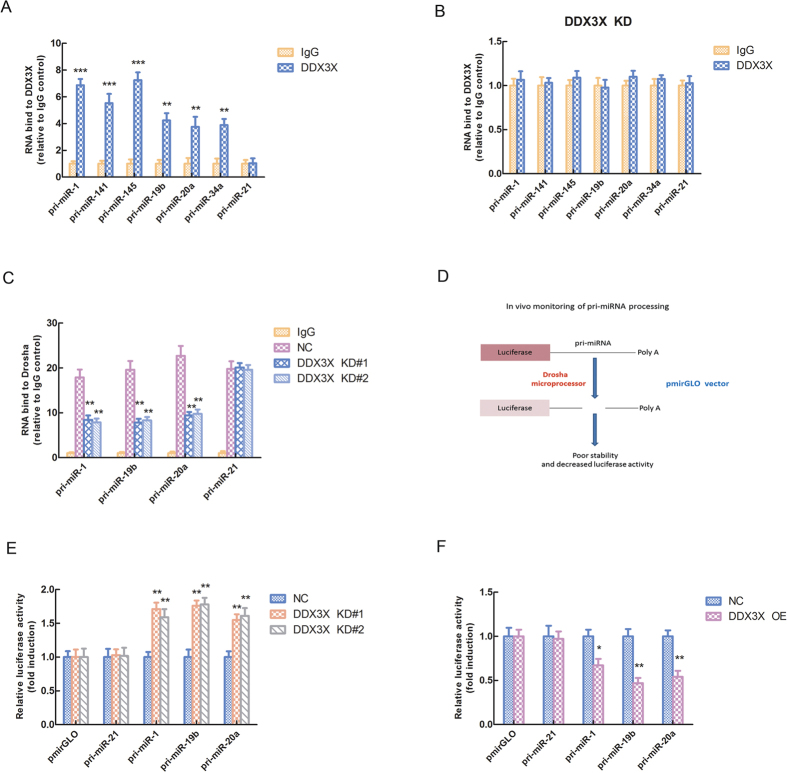
DDX3X facilitates pri-miRNAs binding to Drosha/DGCR8 complex and elevates the processing activity of Drosha/DGCR8 complex on pri-miRNAs. (**A**) The binding affinity of DDX3X-dependent pri-miRNAs with DDX3X tested by RNA immunoprecipitation (RIP) experiment. The DDX3X-independent pri-miRNA (pri-miR-21) was used as an internal control. The asterisk(s) (**^,^***) indicate a significant difference (*p* < 0.01, *p* < 0.001) respectively. (**B**) The binding affinity of DDX3X-dependent pri-miRNAs with DDX3X in DDX3X KD cell lines tested by RNA immunoprecipitation (RIP) experiment. The DDX3X-independent pri-miRNA (pri-miR-21) was used as an internal control. (**C**) The binding affinity of DDX3X-dependent pri-miRNAs with Drosha in DDX3X KD#1, KD#2 and their NC cell lines tested by RNA immunoprecipitation (RIP) experiment. The DDX3X-independent pri-miRNA (pri-miR-21) was used as an internal control. The asterisks (**) indicate a significant difference (*p* < 0.01). (**D**) A schematic model for *in vivo* monitoring of pri-miRNA processing. (**E**) Relative luciferase activities of DDX3X-dependent pri-miRNA pmirGLO vectors in DDX3X KD#1, KD#2 and their NC cell lines tested by dual luciferase reporter assay. The DDX3X-independent pri-miRNA (pri-miR-21) and empty vector pmirGLO were used as internal controls. The asterisks (**) indicate a significant difference (*p* < 0.01). (**F**) Relative luciferase activities of DDX3X-dependent pri-miRNA pmirGLO vectors in DDX3X OE and its NC cell lines tested by dual luciferase reporter assay. The DDX3X-independent pri-miRNA (pri-miR-21) and empty vector pmirGLO were used as internal controls. The asterisk(s) (*^,^**) indicate a significant difference (*p* < 0.05, *p* < 0.01) respectively.

In order to further explore the regulatory mechanisms of DDX3X on miRNA biogenesis, we carried out the RNA immunoprecipitation (RIP) experiment to test the binding affinity of DDX3X-dependent pri-miRNAs with DDX3X. The data showed that DDX3X-dependent pri-miRNAs (pri-miR-1, pri-miR-141, pri-miR-145, pri-miR-19b, pri-miR-20a and pri-miR-34a) could significantly bind to DDX3X compared with IgG control (**p < 0.01, ***p < 0.001) (Fig. 3A). However, when we knocked down the expression of DDX3X, the DDX3X-dependent pri-miRNAs could not bind to DDX3X anymore (Fig. 3B). Moreover, we adopted two DDX3X KD cell lines and used Drosha antibody to conduct RIP assay, so as to pull-down the RNA binding with Drosha. The data indicated that knock-down the expression of DDX3X could significantly down-regulate the pri-miRNA levels which bind to Drosha, compared with NC group (**p < 0.01) (Fig. 3C). And it hinted us that DDX3X could facilitate pri-miRNAs binding to Drosha/DGCR8 complex and set favorable conditions for miRNA processing. In these RIP assays, we used the DDX3X independent pri-miRNA (pri-miR-21) as an internal control to confirm the quality of the system.

To further explore the role of DDX3X in monitoring pri-miRNA processing activity of Drosha/DGCR8 complex, we used the pmirGLO vector to construct pri-miRNA vector, inserting the sequences of pri-miRNA between luciferase gene and poly A tail, and then carry out the dual luciferase reporter assay to test the processing activity of Drosha microprocessor (Fig. 3D). From the schematic model, we can see that if pri-miRNA is processed by Drosha microprocessor, the luciferase gene will have poor stability and present decreased luciferase activity. So the lower level of luciferase activity means the higher level of Drosha microprocessor processing activity. Then we used the DDX3X KD and OE cell lines to conduct the dual luciferase reporter assay. The data demonstrated that the relative luciferase activities of pri-miRNA vectors were significantly up-regulated in DDX3X KD cell lines compared with NC group (**p < 0.01) (Fig. 3E). While, in DDX3X OE cell lines, the relative luciferase activities of pri-miRNA vectors were significantly down-regulated compared with NC group (*p < 0.05, **p < 0.01) (Fig. 3F). All these data suggested that knock-down the expression of DDX3X could inhibit the processing activity of Drosha/DGCR8 complex on pri-miRNAs. Whereas, over-expression of DDX3X could elevate the processing activity of Drosha/DGCR8 complex on pri-miRNAs. In these experiments, we adopted the DDX3X independent pri-miRNA (pri-miR-21) and empty vector pmirGLO as internal controls to maintain the quality of the system.

### The relative expression levels and potential roles of DDX3X-dependent miRNAs (miR-1, miR-141, miR-145, miR-19b, miR-20a and miR-34a) in multiple cancers

In this part, we used the primary data provided in the TCGA (The Cancer Genome Atlas) database to analyze the expression levels of DDX3X-dependent miRNAs in 14 kinds of cancers versus their adjacent normal tissues. These 14 kinds of cancers include urothelial bladder cancer (BLCA), breast cancer (BRCA), colon and rectal adenocarcinoma (CRC), glioblastoma multiforme (GBM), head and neck squamous cell carcinoma (HNSC), chromophobe renal cell carcinoma (KICH), clear cell kidney carcinoma (KIRC), acute myeloid leukemia (LAML), lung adenocarcinoma (LUAD), lung squamous cell carcinoma (LUSC), ovarian serous cystadenocarcinoma (OV), cutaneous melanoma (SKCM), papillary thyroid carcinoma (THCA), and uterine corpus endometrial carcinoma (UCEC). The total case numbers of these 14 cancers were 5613 clinical samples.

**Figure 4:**
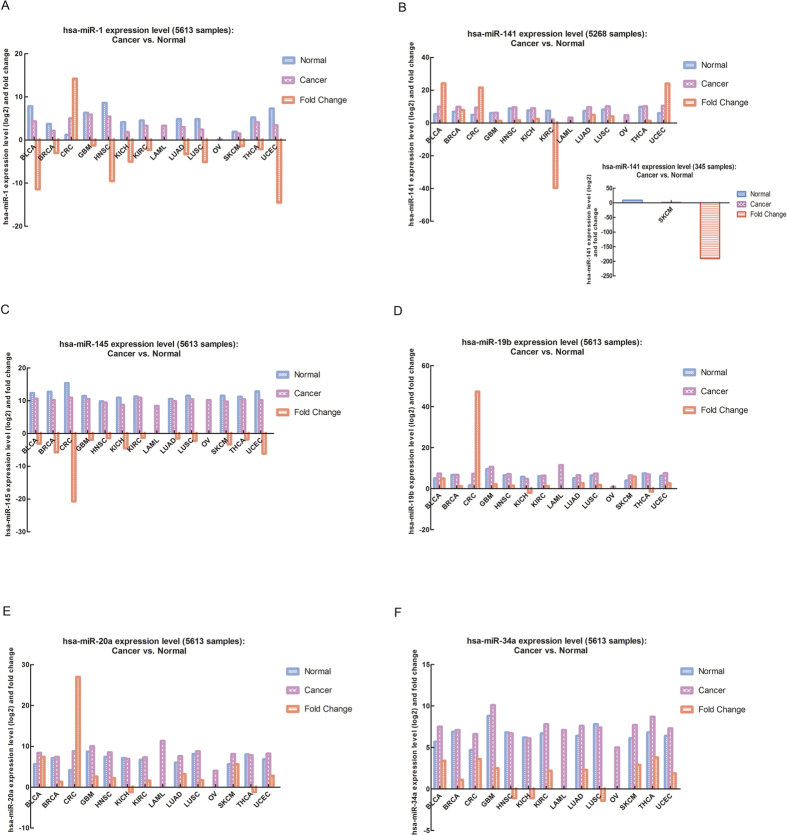
The relative expression levels of DDX3X-dependent miRNAs in 14 kinds of cancers. The expression levels and fold changes of miR-1 (**A**), miR-141 (**B**), miR-145 (**C**), miR-19b (**D**), miR-20a (**E**), miR-34a (**F**) in 14 kinds of cancers versus their adjacent normal tissues analyzed by The Cancer Genome Atlas (TCGA) database and starBase database in 5613 clinical samples. Abbreviations: BLCA: urothelial bladder cancer, BRCA: breast cancer, CRC: colon and rectal adenocarcinoma, GBM: glioblastoma multiforme, HNSC: head and neck squamous cell carcinoma, KICH: chromophobe renal cell carcinoma, KIRC: clear cell kidney carcinoma, LAML: acute myeloid leukemia, LUAD: lung adenocarcinoma, LUSC: lung squamous cell carcinoma, OV: ovarian serous cystadenocarcinoma, SKCM: cutaneous melanoma, THCA: papillary thyroid carcinoma, UCEC: uterine corpus endometrial carcinoma.

For the expression level of miR-1 in these cancers, we can see that miR-1 expression level was significantly up-regulated in CRC, while its expression level was significantly down-regulated in majority of cancer types, such as BLCA, BRCA, GBM, HNSC, KICH, KIRC, LUAD, LUSC, SKCM, THCA, and UCEC (Fig. 4A). These clinical data indicated that miR-1 might play diverse roles in different types of cancers and it could act as either “oncomir” or “tumor suppressive miRNA” to exert its various biological functions. For the expression level of miR-141, it was significantly up-regulated in many cancers, including BLCA, BRCA, CRC, LUAD, LUSC and UCEC, while its expression level was dramatically down-regulated in KIRC and SKCM (Fig. 4B). These data suggested that “dual roles” of miR-141 in multiple cancers were worthwhile doing further researches, especially in KIRC and SKCM.

Using the same strategy and analysis method, we can further analyze the expression levels of other four miRNAs in different cancers. The expression level of miR-145 was significantly down-regulated in almost all the 12 cancer types (Fig. 4C). And the expression patterns of miR-19b and miR-20a exhibited almost the same trend. Their expression levels were down-regulated in KICH and THCA, while in other types of cancers, their expression levels were all significantly up-regulated, especially in CRC (Fig. 4D,E). This is because miR-19b and miR-20a both belong to the miR-17-92 family and share the similarity in miRNA sequences, so their expression patterns and biological functions in cancers might be same. Moreover, the expression level of miR-34a also demonstrated the specificity for different cancer types. In HNSC, KICH, LUSC, miR-34a expression level was down-regulated. Except from these 3 cancers, its expression level was significantly up-regulated in other 9 cancer types (Fig. 4F).

Besides, the correlations between the expression levels of DDX3X and its dependent six miRNAs in different kinds of cancers suggested that DDX3X expression level was widely down-regulated in almost 10 types of cancers (Fig. S1A). And its dependent miR-145 and miR-1 expression levels exhibited the same tendency accordingly. However, for other four miRNAs, their expression levels were in accordance with DDX3X expression level only in some specific types of cancers. For example, miR-141 expression level had the same trend with DDX3X in KIRC and SKCM; miR-19b and miR-20a expression levels shared the same tendency with DDX3X in KICH and THCA; miR-34a expression level was in accordance with DDX3X in HNSC, KICH and LUSC. Whereas, for the expression levels of DDX3X irrelevant miRNAs, such as miR-21 and miR-155, their expression patterns were consistently up-regulated and quite opposite with DDX3X in almost all kinds of cancers (Fig. S1B,C).

Above all, these data hinted that the expression changes of DDX3X-dependent miRNAs might be attributed to DDX3X level change in several human cancers, although it depends on the specificity of cancer types. And all these clinical expression data will provide us new directions and insights for exploring potential roles and biological functions of DDX3X-dependent miRNAs in different cancer types.

### The downstream targets analysis and signaling pathways illustration for the biological functions of DDX3X-dependent miRNAs

**Figure 5:**
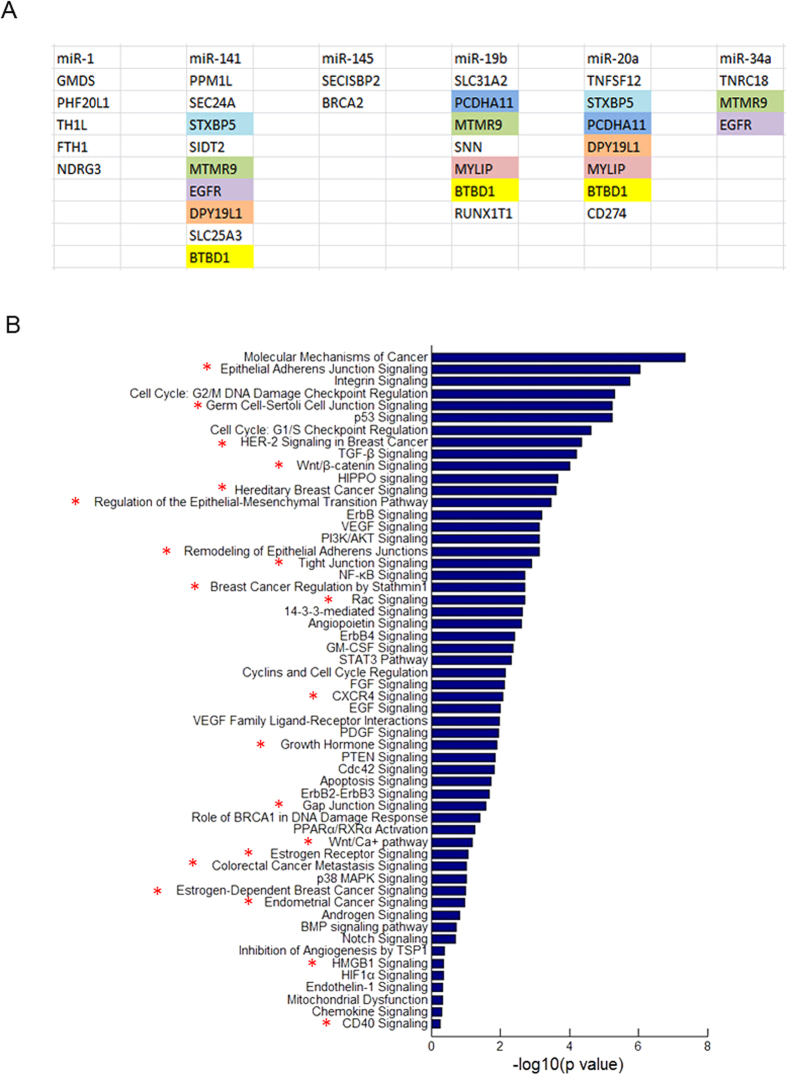
The downstream targets analysis and signaling pathways illustration for the biological functions of DDX3X-dependent miRNAs. (**A**) Prediction the putative downstream targets of six DDX3X-dependent miRNAs by miRBase, miRecords, miRanda, TargetScan, PITA, RNAhybrid, and starBase databases. The key potential downstream targets were summarized and the same targets they shared were highlighted in different colors. (**B**) Systematical analysis of the biological functions and signaling pathways for the downstream targets of DDX3X-dependent miRNAs by the Ingenuity Pathway Analysis (IPA) software. The key signaling pathways were listed according to their ranking scores and the functional similarity pathways were highlighted in red asterisks (*).

In order to deeply study the potential roles of DDX3X-dependent miRNAs in cancers, we used various miRNA downstream targets prediction databases to screen the putative downstream targets of six DDX3X-dependent miRNAs. These prediction databases mainly include miRBase, miRecords, miRanda, TargetScan, PITA, RNAhybrid, and starBase. After comprehensively analyzing and comparing all the data in these databases, we summarized the key potential downstream targets for DDX3X-dependent miRNAs and highlighted the same targets they shared in different colors (Fig. 5A). We can see that for miR-141, miR-19b, miR-20a and miR-34a, they shared part of common targets together. For example, the gene MTMR9 (myotubularin related protein 9) can be targeted by miR-141, miR-19b and miR-34a. The gene BTBD1 (BTB domain containing 1) can be targeted by miR-141, miR-19b and miR-20a. The gene MYLIP (myosin regulatory light chain interacting protein) and PCDHA11 (protocadherin alpha 11) can both be targeted by miR-19b and miR-20a. All these database prediction data suggested that there were complicated targets interaction networks in the downstream of DDX3X-dependent miRNAs and it was worthwhile doing systematic study to analyze their regulatory relationships and exploring their potential functions in cancers.

In the next step, we used the IPA (Ingenuity Pathway Analysis) software to systematically analyze the biological functions and signaling pathways for the downstream targets of DDX3X-dependent miRNAs. And then we drew a figure for the key signaling pathways according to their ranking scores and highlighted the functional similarity pathways in red asterisks (Fig. 5B). The figure showed that in the downstream of DDX3X-dependent miRNAs, most of their target genes were involved in the regulation of cancer invasion, EMT (Epithelial-Mesenchymal Transition) and metastasis processes and their related signaling pathways, such as the Epithelial Adherens Junction Signaling, Wnt/β-catenin Signaling, Regulation of the Epithelial-Mesenchymal Transition Pathway, Remodeling of Epithelial Adherens Junctions, Tight Junction Signaling, Rac Signaling, CXCR4 Signaling, Gap Junction Signaling and so on. Meanwhile, these target genes also participated in the regulation of breast cancer and estrogen-related cancer signalings, such as HER-2 Signaling in Breast Cancer, Hereditary Breast Cancer Signaling, Breast Cancer Regulation by Stathmin1, Estrogen Receptor Signaling, Estrogen-Dependent Breast Cancer Signaling, Endometrial Cancer Signaling and so on. All in all, these bioinformatics analysis data will pave us new avenues for thoroughly studying the biological functions of DDX3X-dependent miRNAs in cancers.

## Discussion

In this paper, we mainly discussed the regulatory mechanism of DDX3X in miRNA biogenesis process and used the bioinformatics database and software to deeply explore the biological functions and potential roles of miRNA downstream target genes, so as to get a general outlook for the DDX3X-dependent miRNA regulatory networks. The most striking finding of this research is that we found DDX3X can act as a novel RNA binding protein to interact with Drosha/DGCR8 complex (miRNA microprocessor), promote pri-miRNA processing activity, and finally elevate mature miRNA expression level. The significance of this study on the upstream of miRNA expression regulation is that the upstream modulators can affect a subset of miRNAs expression levels and influence multiple downstream target genes expression, so as to participate in the process of cancer development more widely and comprehensively compared with single miRNA regulatory model.

Still, in our primary research results, we admit that although DDX3X interacts with Drosha/DGCR8 complex, the DDX3X’s KO/OE strategies seems affect a small part of miRNA expression levels, so it is necessary to explore what are the co-factors leading to such a specificity. Based on this notion, we used the TCGA expression data to investigate the conditional mutual information about the DDX3X and its potential co-factors. The data showed that besides DGCR8, DDX3X could also interact with multiple RNA binding proteins, such as HuR, PTB, FUS, LIN28, MOV10, EWSR1 and so on (Table S1). And the presence or absence of these modulators could effectively affect DDX3X’s ability to regulate the expression levels of its dependent miRNAs. Take DDX3X co-factors FUS and MOV10 as examples, FUS was negatively correlated with DDX3X in SKCM samples (Fig. S2A), so the presence of FUS could down-regulate the expression levels of DDX3X-dependent miRNAs, such as miR-1, miR-141 and miR-145 in SKCM. Whereas, the absence of FUS could otherwise up-regulate the expression levels of these three DDX3X-dependent miRNAs. However, the case for MOV10 is quite opposite. MOV10 was positively correlated with DDX3X in GBM samples (Fig. S2B), so the presence of MOV10 could up-regulate the expression levels of DDX3X-dependent miRNAs, such as miR-1 and miR-145 in GBM. Whereas, the absence of MOV10 could otherwise down-regulate the expression levels of these two DDX3X-dependent miRNAs. So the interactions between DDX3X and its co-factors might contribute to the specificity of miRNA expression regulation in specific kinds of cancers.

Actually, for the modulation of miRNA biogenesis, various key factors also play an important role in this process, apart from the DEAD-box RNA proteins DDX5 (p68), DDX17 (p72) and DDX3X. More specifically, through the mediation of p68 and p72, p53 could affect Drosha/DGCR8 complex processing activity and promote pri-miRNA biogenesis process^23^. However, if the p53 is mutant at C135Y, R175H, R273H and R248Q sites, the binding of mut-p53 with p68/p72 will lead to the dissociation of Drosha/DGCR8 complex and interfere with Drosha and pri-miRNAs association, so as to hinder the processing of pri-miRNAs and result in miRNA deregulation observed in cancers^24^. Moreover, with the assistant of p68, SMADs can also control Drosha-mediated pri-miRNA processing and miRNA maturation^25^. As key signal transducers of TGF-β (transforming growth factor beta) and BMP (bone morphogenetic protein) signaling pathways, SMADs can be recruited to p68/Drosha microprocessor complex and play a critical regulatory role in the nuclear processing of pri-miRNAs by Drosha/DGCR8 complex^26^. Additionally, there is another essential factor ERα (Estrogen Receptor α) in this system, which can negatively modulate miRNA biogenesis process^27^. That is because in the presence of estradiol (E2), it can bind to ERα, activate its activity and further block Drosha processing of a subset of p68/p72-dependent miRNAs, leading to the estrogen receptor-mediated inhibition of nuclear pri-miRNA processing^28^. Besides, the ERα-mediated regulation of miRNA biogenesis will result in the stabilization of mRNAs targeted by those p68/p72-dependent miRNAs and increase target genes expression levels.

Nowadays, using the bioinformatics tools and the big data in the prediction databases to do the miRNA-related research has become a new trend. That is because the regulatory networks for miRNA are rather complicated. One miRNA can be regulated by various transcription factors and can target multiple downstream genes expressions. Meanwhile, one target gene can also be modulated by numerous of miRNAs. So the “transcription factors—miRNAs—target genes” consist of a complex interaction network to affect miRNA-mediated regulation of gene expression together. With the development of bioinformatics technology and under the support of US government, the TCGA database launched by NIH is emerging, which provides us a open database to access the information about gene copy number variations, gene expression levels and survival curves in the clinical samples of multiple kinds of cancers. Base on the primary data from TCGA, several more practical and easy-using databases have been developed, such as SurvExpress, starBase, Kaplan Meier-plotter, Nexus DB and so on. All these useful databases and high-throughput platforms facilitate us to carry out research more directionally and shed light on the clinical significance and prognosis outcome for specific studies on miRNA and its target genes in cancers.

To sums up, in the research field of miRNA biogenesis, there are still lots of things need to be explored and investigated. Besides finding key regulators to modulate the activity of Drosha/DGCR8 microprocessor, we can also interfere with miRNA processing through other steps, such as affecting the functions of Exportin-5 and Dicer/Ago complex, or utilizing the mutant types of critical enzymes and factors participating in miRNA biogenesis process. More importantly, with the aid of bioinformatics tools and newly developed databases, we can analyze the biological functions and signaling pathways of miRNA and its potential target genes more comprehensively and systematically, so as to get a clear and complete picture for the study of basic mechanisms and clinical significances.

## Materials and Methods

### Cell culture

The U2OS and HEK293 cell lines were cultured in DMEM with 10% fetal bovine serum (FBS), 1% glutamine, and 1% antibiotics. The cell lines were grown in a humidified incubator at 37 °C with 5% CO_2_.

### Immunoprecipitation (IP)

The HEK293 cells were lysed on ice for 30 min in IP buffer containing protease inhibitor cocktail. Then the cell lysates (600 μg) were incubated with 3 μg of antibodies or normal IgG at 4 °C overnight with rotary agitation. After that the protein A or G sepharose beads were added to the lysates and incubated for additional 4 hrs at 4 °C with rotary agitation. Then the beads be washed by IP buffer three times, resuspended in 3% SDS sample buffer and boiled for 10 min at 100 °C. The total cell lysates and immunoprecipitates were separated by SDS-PAGE gel and analyzed by Western blot. The images were processed by Image Lab 4.0 (Bio-Rad Laboratories, Inc.) software packages. The antibodies used in this experiment were as follows: anti-DDX3X antibody (A300–474A, Bethyl Laboratories, USA), anti-Drosha antibody (A301–886A, Bethyl Laboratories, USA), anti-DGCR8 antibody (A302–468A, Bethyl Laboratories, USA), anti-Flag M2 antibody (F3165, Sigma, USA), anti-p84 antibody (ab131268, Abcam, USA), anti-Dicer antibody (ab13502, Abcam, USA), anti-β-actin antibody (I-19) (sc-1616, Santa Cruz, USA).

### RNA immunoprecipitation (RIP)

RIP assay was performed as the previous published papers described^29^,^30^. Briefly, the cells were crosslinked with 1% formaldehyde for 20 min, and cell pellets were resuspended in prepared buffer (1% SDS, 10 mM EDTA, 50 mM Tris-HCl (pH 8.1), protease inhibitor, 50 U/ml RNase inhibitor). After incubated in ice for 10 min, the cell pellets were disrupted by sonication, and the lysates were subjected to immunoprecipitation with control IgG or anti-DDX3X and anti-Drosha antibody. Then it followed by the processes of stringent washing, elution, and reversal of crosslinking. Finally, the RNA was resuspended in 20 μl of TE buffer and incubated with DNase I for 30 min at 37 °C to remove remaining DNA. After extraction with phenol:chloroform:isoamyl alcohol (25:24:1), RNA was precipitated with ethanol and dissolved in 20 μl of DEPC-treated water. After that, the RNA sample (5 μl) was used for the RT-PCR.

### Pri-miRNA processing activity assay

The pri-miRNA processing activity assay was performed as the previous published paper described^31^. First, the pmirGLO expression vectors for pri-miR-1, pri-miR-19b, pri-miR-20a, and pri-miR-21 were constructed. Then, they were transfected into DDX3X KD or OE and their negative control (NC) cells. 48 hrs after that, the dual luciferase reporter assay was carried out and the ratios of Firefly and Renilla luciferase were obtained using the Dual-Luciferase Reporter Assay Kit (Cat#: E1910, Promega, USA). In this system, the empty pmirGLO vector was used as an internal control. Firefly luciferase values were normalized to Renilla, and the ratios of Firefly/Renilla activity were presented.

### Real-time PCR assays

The mature miRNAs quantitative real-time PCR assays were performed using TaqMan^®^ MicroRNA Assays (Applied Biosystems, USA), and U6 snRNA (Applied Biosystems, USA) was used as an internal control. For the pri-miRNAs and DDX3X quantitative real-time PCR assays, GAPDH was used as an internal control. And their expression levels were detected using SYBR Green I chemistry (Power SYBR Green PCR Master Mix, ABI Inc., USA). Real-time PCR and data collection were performed with an ABI 7500 sequence detection system. The relative expression levels were calculated using the 2^−△△Ct^ method. The primers were as follows:

*DDX3X*: Forward: 5′-GGAGGAAGTACAGCCAGCAAAG-3′

Reverse: 5′-CTGCCAATGCCATCGTAATCACTC-3′

*GAPDH*: Forward: 5′-AGCCACATCGCTCAGACAC-3′

Reverse: 5′-GCCCAATACGACCAAATCC-3′

### Analysis of predicted miRNAs targets

The putative targets of miRNAs were analyzed using the following six respective databases: miRecords (http://mirecords.biolead.org/), miRanda (http://www.microrna.org//miranda.html), TargetScan (http://genes.mit.edu/targetscan), PITA (http://genie.weizmann.ac.il/pubs/mir07/mir07_data.html), RNAhybrid (http://bibiserv.techfak.uni-bielefeld.de/rnahybrid/), and starBase (http://starbase.sysu.edu.cn/).

### MiRNA expression level analysis and downstream targets biological functions illustration

The expression levels of DDX3X-dependent miRNAs in 14 kinds of cancers versus their adjacent normal tissues were analyzed by The Cancer Genome Atlas (TCGA) database and starBase database in 5613 clinical samples. Their downstream targets biological functions illustration was made by the Ingenuity Pathway Analysis (IPA) software and the key signaling pathways were highlighted according to their ranking scores and functional similarities.

### Statistical analysis

All quantitative data were expressed as mean values ± S.D. of at least 3 independent experiments. Statistical analysis was performed using SPSS17.0. Significant differences between two groups were compared using the Student’s t-test, and comparisons among more than two groups were performed using analysis of variance (ANOVA). *p-*values < 0.05 were considered to be statistically significant.

## Additional Information

**How to cite this article**: Zhao, L. *et al*. DDX3X promotes the biogenesis of a subset of miRNAs and the potential roles they played in cancer development. *Sci. Rep.* **6**, 32739; doi: 10.1038/srep32739 (2016).

## Supplementary Material

Supplementary Information
