# Supplementary material for: DDX3X promotes the biogenesis of a subset of miRNAs and the potential roles they played in cancer development
Source: Sci Rep. 2016 Sep 2;6:32739. doi: 10.1038/srep32739 (PMC5009351; doi:10.1038/srep32739)
Supplement: Supplementary Information [file srep32739-s1.pdf]

Supplementary information

DDX3X promotes the biogenesis of a subset of miRNAs and the potential roles they played in cancer development

Luqing Zhao[1,2,3*], Yitao Mao[4], Yuelong Zhao[5], Yanong He[5]

[1] Department of Pathology, Xiangya Hospital, Central South University, Changsha, Hunan 410008, China; and Department of Pathology, School of Basic Medical Science, Xiangya School of Medicine, Central South University, Changsha, Hunan 410013, China.

[2] Department of Dermatology, Xiangya Hospital, Central South University, Changsha, Hunan 410008, China.

[3] Hunan Key Laboratory of Skin Cancer and Psoriasis, Xiangya Hospital, Central South University, Changsha, Hunan 410008, China.

[4] Department of Radiology, Xiangya Hospital, Central South University, Changsha, Hunan 410008, China.

[5] School of Computer Science and Engineering, South China University of Technology, Guangzhou, Guangdong 510640, China.

*Correspondence to:

Dr. Luqing Zhao, E-mail: Lqz2010@hotmail.com

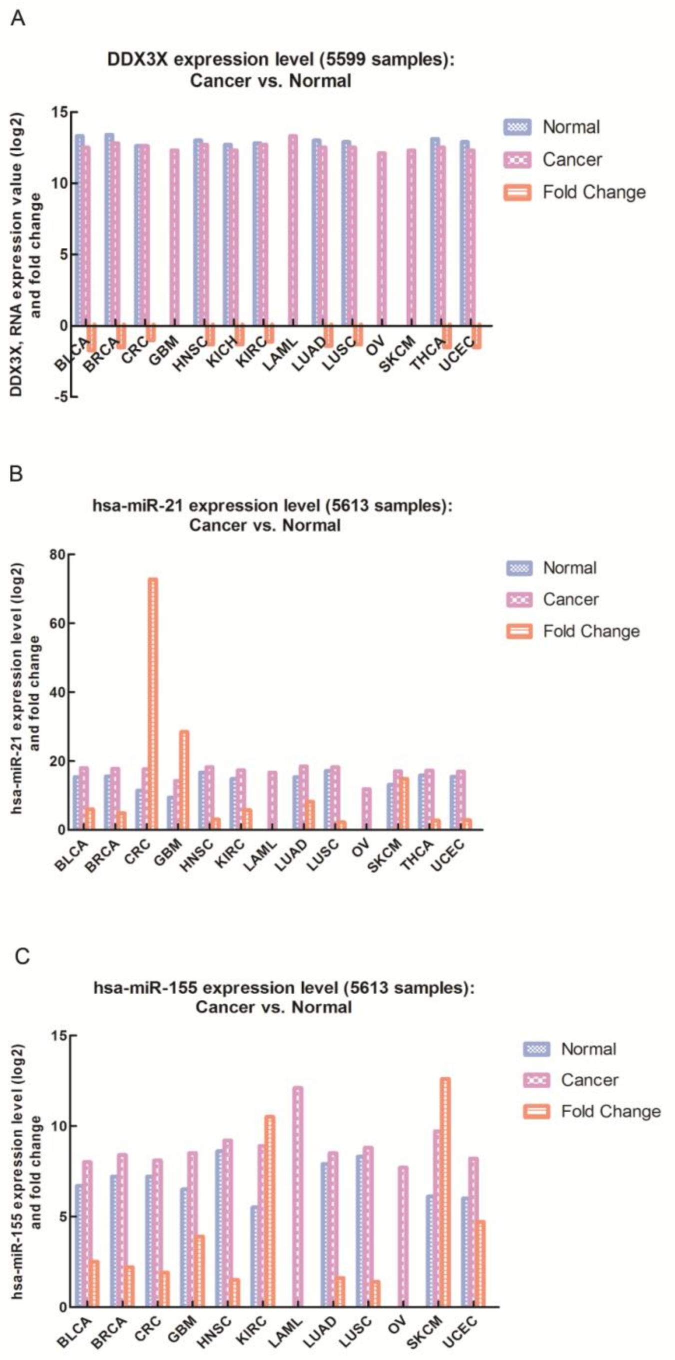

Figure S1. The relative expression levels of DDX3X and its irrelevant miRNAs in different kinds of cancers. The expression levels and fold changes of DDX3X (A), miR-21 (B), miR-155 (C) in different kinds of cancers versus their adjacent normal tissues analyzed by The Cancer Genome Atlas (TCGA) database and starBase database in clinical samples.

Abbreviations: BLCA: urothelial bladder cancer, BRCA: breast cancer, CRC: colon and rectal adenocarcinoma, GBM: glioblastoma multiforme, HNSC: head and neck squamous cell carcinoma, KICH: chromophobe renal cell carcinoma, KIRC: clear cell kidney carcinoma, LAML: acute myeloid leukemia, LUAD: lung adenocarcinoma, LUSC: lung squamous cell carcinoma, OV: ovarian serous cystadenocarcinoma, SKCM: cutaneous melanoma, THCA: papillary thyroid carcinoma, UCEC: uterine corpus endometrial carcinoma.

A

SKCM

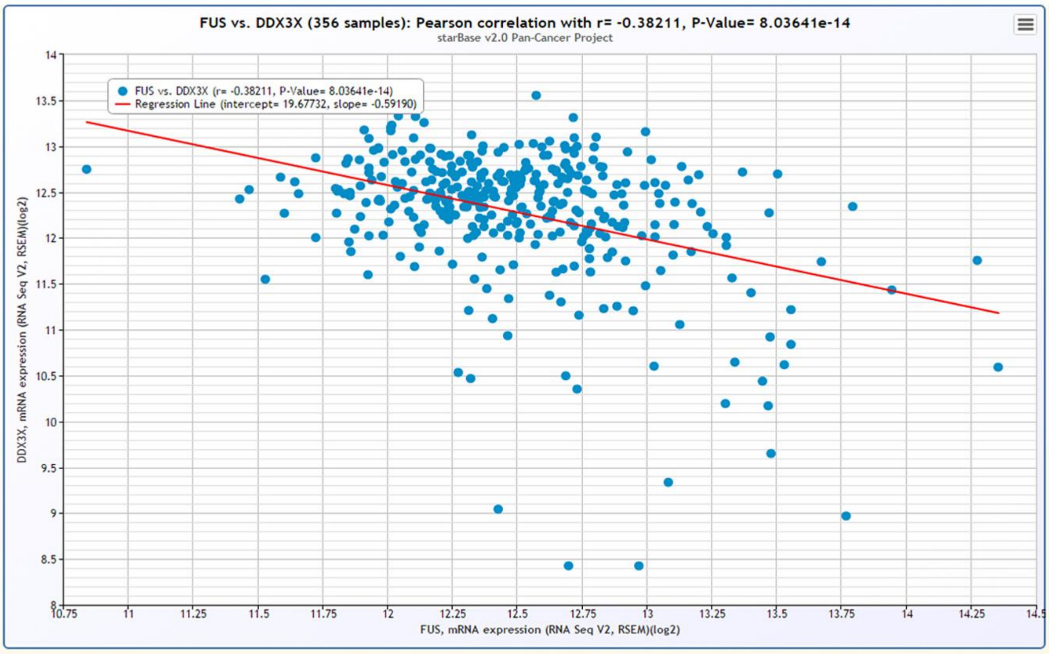

B

GBM

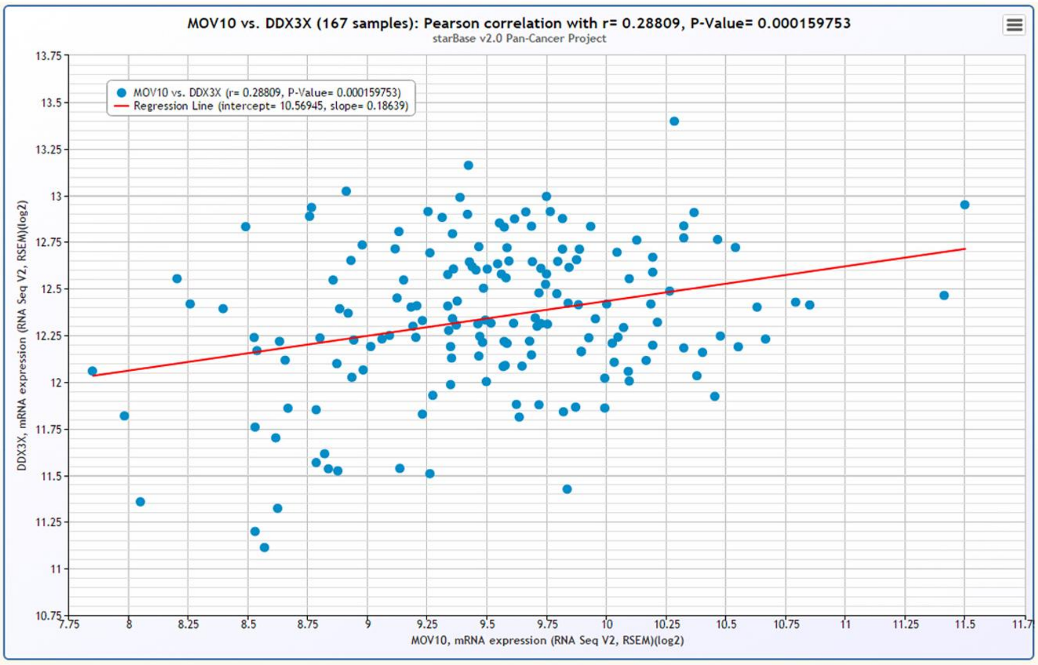

Figure S2. The representative figures for the correlations of DDX3X and its co-factors in cancers. (A) The correlation of DDX3X and its co-factor FUS in 356

SKCM (cutaneous melanoma) samples. (B) The correlation of DDX3X and its co-factor MOV10 in 167 GBM (glioblastoma multiforme) samples. The figures were downloaded from the starBase database and all the data were analyzed by The Cancer Genome Atlas (TCGA) database.

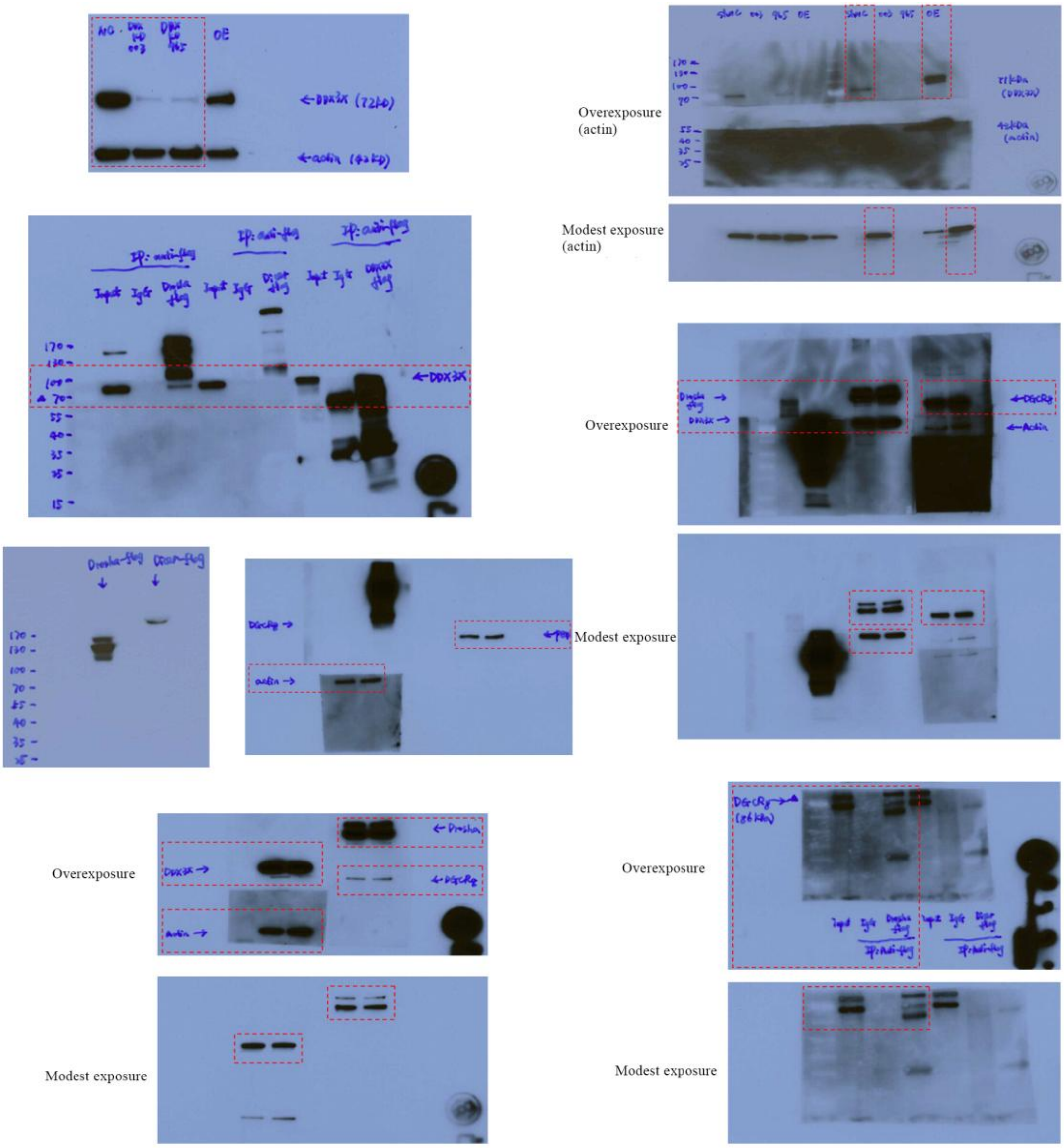

Figure S3. The uncropped full-length blots images for WB and IP experiments.

Table S1. The co-factors which have interactions with DDX3X

Name	Description
HuR	ELAV like RNA binding protein 1
PTB	polypyrimidine tract binding protein 1
IGF2BP1	insulin like growth factor 2 mRNA binding protein 1
IGF2BP2	insulin like growth factor 2 mRNA binding protein 2
IGF2BP3	insulin like growth factor 2 mRNA binding protein 3
eIF4AIII	eukaryotic translation initiation factor 4A3
DGCR8	DGCR8 microprocessor complex subunit
FMRP	fragile X mental retardation protein
FXR1	FMR1 autosomal homolog 1
FXR2	FMR1 autosomal homolog 2
FUS	FUS RNA binding protein
LIN28A	lin-28 homolog A
LIN28B	lin-28 homolog B
MOV10	Mov10 RISC complex RNA helicase
ALKBH5	alkB homolog 5, RNA demethylase
C17ORF85	nuclear cap binding subunit 3
C22ORF28	RNA 2',3'-cyclic phosphate and 5'-OH ligase

CAPRIN1	cell cycle associated protein 1
ZC3H7B	zinc finger CCCH-type containing 7B
EWSR1	EWS RNA binding protein 1
SFRS1	serine and arginine rich splicing factor 1
U2AF65	U2 small nuclear RNA auxiliary factor 2
TIA1	TIA1 cytotoxic granule-associated RNA binding protein
TIAL1	TIA1 cytotoxic granule-associated RNA binding protein-like 1
hnRNPC	heterogeneous nuclear ribonucleoprotein C (C1/C2)
UPF1	UPF1, RNA helicase and ATPase
